# Supplementary material for: A reference tissue implementation of Simultaneous Multifactor Bayesian Analysis (SiMBA) of PET time activity curve data
Source: Imaging Neurosci (Camb). 2025 Nov 14;3:IMAG.a.1011. doi: 10.1162/IMAG.a.1011 (PMC12620908; doi:10.1162/IMAG.a.1011)
Supplement: Supplementary Material [file IMAG.a.1011_supp.pdf]

1 SUPPLEMENTARY MATERIALS

1.1 Supplementary Materials S1: Model Definitions and Analytical Solutions

The Feng model for the AIF is defined as follows:

$$\mathrm{Feng}(t) = \begin{cases} 0 & t \leq t_0 \\ [A(t-t_0) - B - C]\, e^{-\alpha(t-t_0)} + B \cdot e^{-\beta(t-t_0)} + C \cdot e^{-\gamma(t-t_0)} & t > t_0 \end{cases} \tag{1}$$

with free parameters A, B, C, α, β, γ and t_0.

To derive the estimated reference tissue TAC, $C_R(t)$, the hypothetical AIF described by the Feng model, $\mathrm{Feng}(t)$, is convolved with a hypothetical 1TC IRF, IRF

$$\mathrm{IRF_R}(t) = \phi_1 e^{-\theta_1 t} \tag{2}$$

$$C_R(t) = \mathrm{Feng}(t) \otimes \mathrm{IRF_R}(t) \tag{3}$$

$$\tag{4}$$

where the 1TC IRF free parameters are ϕ_1 and θ_1 following the terminology of Gunn, Gunn Cunningham (2001).

The analytical solution of this convolution is as follows:

$$C_R(t) = \phi_1 e^{-t\theta_1} \left(\frac{A\left(e^{t(\theta_1-\alpha)}(-\alpha t + t\theta_1 - 1) + 1\right)}{(\alpha-\theta_1)^2} + \frac{B\left(e^{t(\theta_1-\alpha)} - 1\right)}{\alpha-\theta_1} + \frac{C\left(e^{t(\theta_1-\alpha)} - 1\right)}{\alpha-\theta_1} + \frac{B\left(e^{t(\theta_1-\beta)} - 1\right)}{\theta_1-\beta} + \frac{C\left(e^{t(\theta_1-\gamma)} - 1\right)}{\theta_1-\gamma} \right) \tag{5}$$

This function is fit to all reference tissue TACs to define a parametric representation of these curves which can be entered into the PK model.

For both the FRTM and SRTM PK models, the convolution within the model is of $C_R(t)$ with an exponential decay function whose decay is a property of the other parameters of the model, here defined as c, d and q.

$$C_{\mathrm{T,\,FRTM}}(t) = R_1 \left[C_R'(t) + aC_R(t) \otimes e^{-ct} + bC_R(t) \otimes e^{-dt} \right] \tag{6}$$

$$C_{\mathrm{T,\,SRTM}}(t) = R_1 C_R(t) + pC_R(t) \otimes e^{-qt} \tag{7}$$

In order to create a general analytical solution of the reference tissue model, we solved the convolution between the reference tissue model with a general exponential decay function which we call $\mathrm{ED}(t)$ with rate λ.

$$\mathrm{ED}(t) = e^{-\lambda t} \tag{8}$$

The solution to the convolution of these two functions is then described as follows:

$$
\begin{aligned}
(C_R \otimes \mathrm{ED})(t) = \phi_1 e^{-\lambda t} \Bigg(& \frac{A\left(e^{t(\lambda-\alpha)}-1\right)}{(\alpha-\lambda)(\alpha-\theta_1)^2} + \frac{A\theta_1\left(e^{t(\lambda-\alpha)}(-\alpha t+\lambda t-1)+1\right)}{(\alpha-\lambda)^2(\alpha-\theta_1)^2} \\
& + \frac{A\left(e^{t(\lambda-\theta_1)}-1\right)}{(\alpha-\theta_1)^2(\lambda-\theta_1)} + \frac{A\alpha\left(e^{t(\lambda-\alpha)}(\alpha t-\lambda t+1)-1\right)}{(\alpha-\lambda)^2(\alpha-\theta_1)^2} + \frac{B\left(e^{t(\lambda-\alpha)}-1\right)}{(\lambda-\alpha)(\alpha-\theta_1)} \\
& + \frac{B\left(e^{t(\lambda-\theta_1)}-1\right)}{(\theta_1-\alpha)(\lambda-\theta_1)} + \frac{C\left(e^{t(\lambda-\alpha)}-1\right)}{(\lambda-\alpha)(\alpha-\theta_1)} + \frac{C\left(e^{t(\lambda-\theta_1)}-1\right)}{(\theta_1-\alpha)(\lambda-\theta_1)} + \frac{B\left(e^{t(\lambda-\beta)}-1\right)}{(\beta-\lambda)(\beta-\theta_1)} \\
& - \frac{B\left(e^{t(\lambda-\theta_1)}-1\right)}{(\theta_1-\beta)(\lambda-\theta_1)} + \frac{C\left(e^{t(\lambda-\gamma)}-1\right)}{(\gamma-\lambda)(\gamma-\theta_1)} - \frac{C\left(e^{t(\lambda-\theta_1)}-1\right)}{(\theta_1-\gamma)(\lambda-\theta_1)} \Bigg)
\end{aligned} \tag{9}
$$

1.2 Supplementary Materials S2: Examples of Reference Tissue Fits

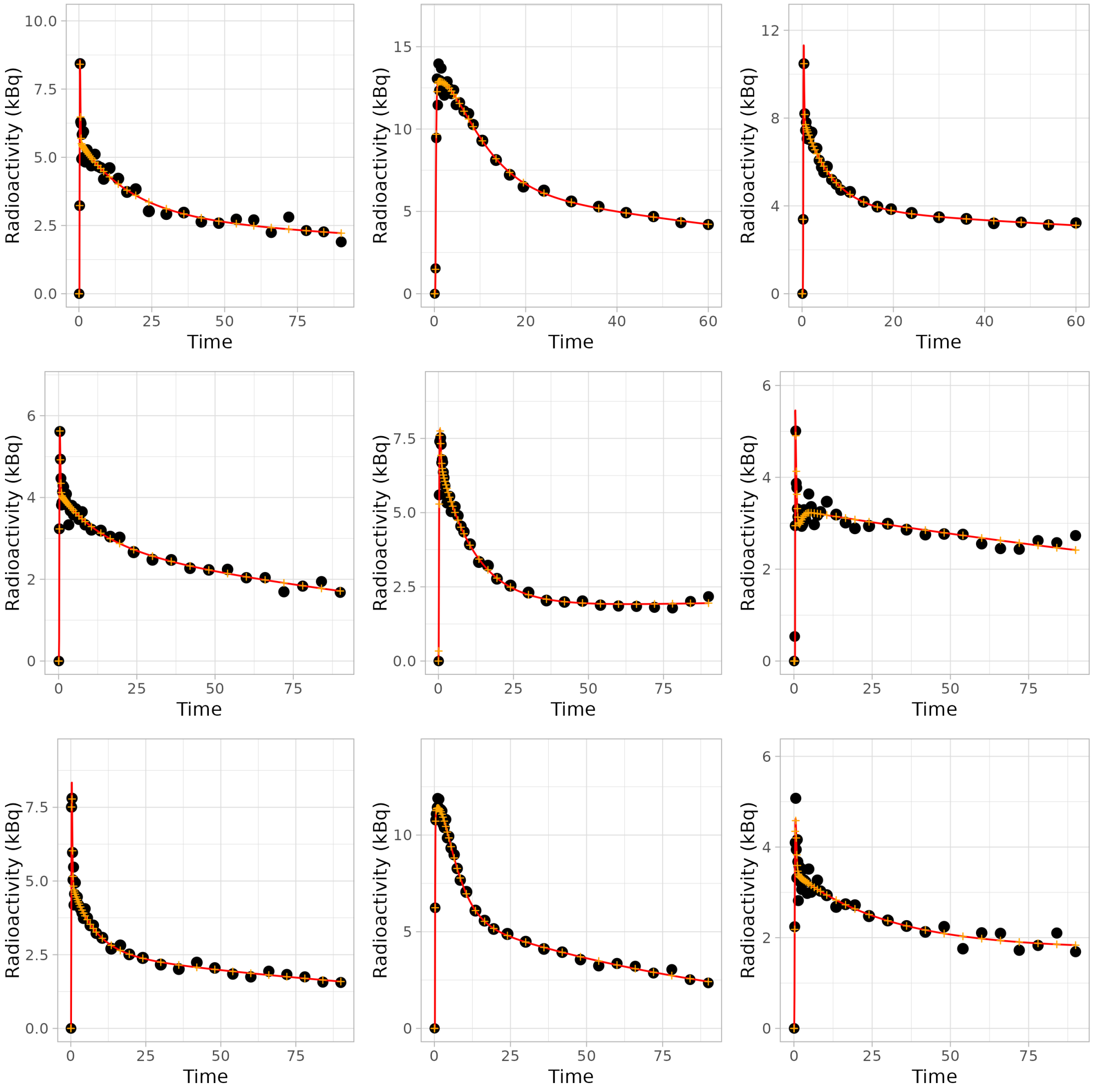

Figure S1. Examples of fits to the cerebellar reference region time activity curves from nine randomly sampled measurements. The size of the data points represents their assigned model weights, and the yellow crosses represent the instantaneous estimated $C_{\mathrm{REF}}(t)$ at the mid-frame time points which are used in the PK model functions.

1.3 Supplementary Materials S3: Prior Definitions

1.3.1 Global Intercepts

Below are the priors defined for the global intercepts. Note that all priors are defined over the natural logarithms of the parameters.

$$
\begin{aligned}
\alpha_{\mathrm{R_1}} &\sim \mathrm{Normal}(0, 0.25)\\
\alpha_{\mathrm{k_2'}} &\sim \mathrm{Normal}(-2, 0.25)\\
\alpha_{\mathrm{BP_{ND}}} &\sim \mathrm{Normal}(0, 0.25)
\end{aligned}
$$

The priors for $\alpha_{\mathrm{R_1}}$ and $\alpha_{\mathrm{BP_{ND}}}$ are defined for the frontal cortex as the reference level of the dummy variable.

1.3.2 Individual deviations

Differences between individuals were defined by specifying the primary pharmacokinetic parameters in one variance-covariance matrix.

$$
\begin{aligned}
\begin{bmatrix}\tau_{\mathrm{R_1}}\\ \tau_{\mathrm{k_2'}}\\ \tau_{\mathrm{BP_{ND}}}\end{bmatrix} &\sim \mathrm{MVNormal}\left(\begin{bmatrix}0\\0\\0\end{bmatrix}, \Sigma_{\mathrm{Subject}}\right)\\
\Sigma_{\mathrm{Subject}} &= \begin{bmatrix}\sigma_{\mathrm{R_1}} & 0 & 0\\ 0 & \sigma_{\mathrm{k_2'}} & 0\\ 0 & 0 & \sigma_{\mathrm{BP_{ND}}}\end{bmatrix} \mathbf{R}_{\mathrm{Subject}} \begin{bmatrix}\sigma_{\mathrm{R_1}} & 0 & 0\\ 0 & \sigma_{\mathrm{k_2'}} & 0\\ 0 & 0 & \sigma_{\mathrm{BP_{ND}}}\end{bmatrix}\\
\sigma_{\mathrm{R_1}} &\sim \text{Half-Normal}(0, 0.3)\\
\sigma_{\mathrm{k_2'}} &\sim \text{Half-Normal}(0, 0.1)\\
\sigma_{\mathrm{BP_{ND}}} &\sim \text{Half-Normal}(0, 0.3)\\
\mathbf{R}_{\mathrm{Subject}} &\sim \mathrm{LKJ}(1)
\end{aligned}
$$

1.3.3 Regional deviations

For $\log BP_{\mathrm{ND}}$ and $\log R_1$, regional differences were defined as unpooled effects using a dummy (indicator) variable defined with reference to the dorsolateral prefrontal cortex. For simplicity, all regional differences were defined as zero-centred regularising priors with the same SD.

$$
\begin{aligned}
\upsilon_{\mathrm{j,R_1}} &\sim \mathrm{Normal}(0, 0.3)\\
\upsilon_{\mathrm{j,BP_{ND}}} &\sim \mathrm{Normal}(0, 0.3)
\end{aligned}
$$

For $\mathrm{k_2'}$, regional differences were defined as pooled variables, arising from a common distribution

$$
\begin{aligned}
\upsilon_{\mathrm{k_2'}} &\sim \mathrm{Normal}(0, \sigma_{\mathrm{k_2'}})\\
\sigma_{\mathrm{k_2'}} &\sim \text{Half-Normal}(0, 0.1)
\end{aligned}
$$

1.3.4 TAC deviations

For the Individual $\times$ Region deviations, we made use of highly-constrained deviations

$$
\begin{bmatrix} \phi_{\mathrm{R}_1} \\ \phi_{\mathrm{k}_2'} \\ \phi_{\mathrm{BP}_{\mathrm{ND}}} \end{bmatrix} \sim \mathrm{MVNormal}\left(\begin{bmatrix} 0 \\ 0 \\ 0 \end{bmatrix}, \Sigma_{\mathrm{TAC}} \right)
$$

$$
\Sigma_{\mathrm{TAC}} = \begin{bmatrix} \sigma_{\mathrm{R}_1} & 0 & 0 \\ 0 & \sigma_{\mathrm{k}_2'} & 0 \\ 0 & 0 & \sigma_{\mathrm{BP}_{\mathrm{ND}}} \end{bmatrix} \mathbf{R}_{\mathrm{TAC}} \begin{bmatrix} \sigma_{\mathrm{R}_1} & 0 & 0 \\ 0 & \sigma_{\mathrm{k}_2'} & 0 \\ 0 & 0 & \sigma_{\mathrm{BP}_{\mathrm{ND}}} \end{bmatrix}
$$

$$
\begin{aligned}
\sigma_{\mathrm{R}_1} &\sim \text{Half-Normal}(0\,,\,0.025) \\
\sigma_{\mathrm{k}_2'} &\sim \text{Half-Normal}(0\,,\,0.025) \\
\sigma_{\mathrm{BP}_{\mathrm{ND}}} &\sim \text{Half-Normal}(0\,,\,0.025) \\
\mathbf{R}_{\mathrm{TAC}} &\sim \mathrm{LKJ}(2)
\end{aligned}
$$

1.3.5 Covariates

Covariate effects were all estimated using zero-centred regularising priors. For the assessment of age, we defined the following priors, for centred age scaled so that a unit change represents a decade.

$$
\begin{aligned}
\beta_{\mathrm{BP}_{\mathrm{ND}},\mathrm{Age}} &\sim \mathrm{Normal}(\,0\,,\,0.1\,) \\
\beta_{\mathrm{k}_2',\mathrm{Age}} &\sim \mathrm{Normal}(\,0\,,\,0.1\,)
\end{aligned}
$$

Clinical covariates were defined with wider priors

$$
\beta_{\mathrm{BP}_{\mathrm{ND}},\mathrm{Clinical}} \sim \mathrm{Normal}(\,0\,,\,0.2\,)
$$

using the same prior for MDD-Control and Treatment-Baseline (ECT, ketamine and placebo) contrasts, as well as for the centred change in symptom scores scaled to a ΔHAM-D of 10 points.

The random variation in slopes between regions was defined using the mean estimate and random slopes derived from a common distribution using the following priors for age:

$$
\begin{aligned}
\beta_{k,\mathrm{BP}_{\mathrm{ND}},\mathrm{Age}} &\sim \beta_{\mathrm{BP}_{\mathrm{ND}},\mathrm{Age}} + \mathrm{Normal}(0\,,\,\sigma_{\mathrm{BP}_{\mathrm{ND}},\mathrm{Age}}) \\
\sigma_{\mathrm{BP}_{\mathrm{ND}},\mathrm{Age}} &\sim \text{Half-Normal}(0\,,\,0.05)
\end{aligned}
$$

and for clinical covariates:

$$
\begin{aligned}
\beta_{k,\mathrm{BP}_{\mathrm{ND}},\mathrm{Clinical}} &\sim \beta_{\mathrm{BP}_{\mathrm{ND}},\mathrm{Clinical}} + \mathrm{Normal}(0\,,\,\sigma_{\mathrm{BP}_{\mathrm{ND}},\mathrm{Clinical}}) \\
\sigma_{\mathrm{BP}_{\mathrm{ND}},\mathrm{Clinical}} &\sim \text{Half-Normal}(0\,,\,0.1)
\end{aligned}
$$

To account for differences between centres, we made use of priors for overall parameter mean shifts, with one parameter estimated for differences to each other centre (i.e. using KI as the reference centre, we estimated a deviation each for both the NRU and NMS datasets).

$$
\begin{aligned}
\beta_{\mathrm{R}_1,\mathrm{Centre}} &\sim \mathrm{Normal}(\,0\,,\,0.1\,) \\
\beta_{\mathrm{k}_{2'},\mathrm{Centre}} &\sim \mathrm{Normal}(\,0\,,\,0.1\,) \\
\beta_{\mathrm{BP}_{\mathrm{ND}},\mathrm{Centre}} &\sim \mathrm{Normal}(\,0\,,\,0.1\,)
\end{aligned}
$$

For R_1 and BP_{ND}, we also defined Region × Centre interaction effects to account for differences at the region-within-centre level, with the following priors for each region and centre.

$$\beta_{R_1,\text{Centre}\times\text{Region}} \sim \text{Normal}(0, 0.1)$$
$$\beta_{BP_{ND},\text{Centre}\times\text{Region}} \sim \text{Normal}(0, 0.1)$$

In R code using the `brms` package, the code for defining the model equation and priors is as follows:

```
formula <- bf( TAC ~ srtm_model(logR1, logk2prime, logBPnd,
                                 t_tac, t0,
                                 A, B, C,
                                 alpha, beta, gamma,
                                 Ph1, Th1),
               lf(sigma ~ 1 + s(t_tac, by=Centre) +
                    Centre +
                    InjRad_logc +
                    dur_logc +
                    logRegSize_c +
                    (0 + logRegSize_c | Centre) +
                    (1 | Region:Centre) +
                    (1 | PET), center = FALSE),
               # Nonlinear variables
               logR1 ~ 1 + Centre*Region + (1|k|ID) +
                 (1|1|PET:Region),
               logk2prime ~ 1 + Age_dec_c + Centre + (1|m|Region) +
                 (1|k|ID) + (1|1|PET:Region),
               logBPnd ~ 1 + Centre*Region +
                 Age_dec_c + (0 + Age_dec_c | Region) +
                 DiagnosisMDD + (0 + DiagnosisMDD | Region) +
                 TreatmentPlacebo +
                 TreatmentKetamine + (0 + TreatmentKetamine | Region) +
                 TreatmentECT + (0 + TreatmentECT | Region) +
                 delta_Symptoms +
                 (1|k|ID) + (1|1|PET:Region),
               # Nonlinear fit
               nl = TRUE, center = TRUE)

mlsrtm_prior <- c(

  set_prior("normal(0, 0.25)", nlpar = "logR1"),
  set_prior("normal(-2, 0.25)", nlpar = "logk2prime"),
  set_prior("normal(0, 0.25)", nlpar = "logBPnd"),

  set_prior("normal(0, 0.3)", nlpar = "logR1", class = "sd", group="ID"),
  set_prior("normal(0, 0.1)", nlpar = "logk2prime", class = "sd", group="ID"),
  set_prior("normal(0, 0.3)", nlpar = "logBPnd", class = "sd", group="ID"),

  set_prior("normal(0, 0.025)", nlpar = "logR1", class = "sd", group="PET:Region"),
  set_prior("normal(0, 0.025)", nlpar = "logk2prime", class = "sd", group="PET:Region"
    ),
  set_prior("normal(0, 0.025)", nlpar = "logBPnd", class = "sd", group="PET:Region"),

  set_prior("normal(0, 0.1)", nlpar = "logk2prime", class = "sd", group="Region"),

  set_prior("normal(0, 0.3)", coef="RegionACC",     nlpar="logR1"),
  set_prior("normal(0, 0.3)", coef="RegionAMG",     nlpar="logR1"),
  set_prior("normal(0, 0.3)", coef="RegionDBS",     nlpar="logR1"),
  set_prior("normal(0, 0.3)", coef="RegionHIP",     nlpar="logR1"),
  set_prior("normal(0, 0.3)", coef="RegionINS",     nlpar="logR1"),
  set_prior("normal(0, 0.3)", coef="RegionOC",      nlpar="logR1"),
  set_prior("normal(0, 0.3)", coef="RegionTHA",     nlpar="logR1"),
  set_prior("normal(0, 0.3)", coef="RegionVSTR",    nlpar="logR1"),

  set_prior("normal(0, 0.1)", coef="CentreNRU:RegionACC",     nlpar="logR1"),
  set_prior("normal(0, 0.1)", coef="CentreNRU:RegionAMG",     nlpar="logR1"),
  set_prior("normal(0, 0.1)", coef="CentreNRU:RegionDBS",     nlpar="logR1"),
```

```
set_prior("normal(0, 0.1)", coef="CentreNRU:RegionHIP",     nlpar="logR1"),
set_prior("normal(0, 0.1)", coef="CentreNRU:RegionINS",     nlpar="logR1"),
set_prior("normal(0, 0.1)", coef="CentreNRU:RegionOC",      nlpar="logR1"),
set_prior("normal(0, 0.1)", coef="CentreNRU:RegionTHA",     nlpar="logR1"),
set_prior("normal(0, 0.1)", coef="CentreNRU:RegionVSTR",    nlpar="logR1"),

set_prior("normal(0, 0.1)", coef="CentreNMS:RegionACC",     nlpar="logR1"),
set_prior("normal(0, 0.1)", coef="CentreNMS:RegionAMG",     nlpar="logR1"),
set_prior("normal(0, 0.1)", coef="CentreNMS:RegionDBS",     nlpar="logR1"),
set_prior("normal(0, 0.1)", coef="CentreNMS:RegionHIP",     nlpar="logR1"),
set_prior("normal(0, 0.1)", coef="CentreNMS:RegionINS",     nlpar="logR1"),
set_prior("normal(0, 0.1)", coef="CentreNMS:RegionOC",      nlpar="logR1"),
set_prior("normal(0, 0.1)", coef="CentreNMS:RegionTHA",     nlpar="logR1"),
set_prior("normal(0, 0.1)", coef="CentreNMS:RegionVSTR",    nlpar="logR1"),

set_prior("normal(0, 0.3)", coef="RegionACC",    nlpar="logBPnd"),
set_prior("normal(0, 0.3)", coef="RegionAMG",    nlpar="logBPnd"),
set_prior("normal(0, 0.3)", coef="RegionDBS",    nlpar="logBPnd"),
set_prior("normal(0, 0.3)", coef="RegionHIP",    nlpar="logBPnd"),
set_prior("normal(0, 0.3)", coef="RegionINS",    nlpar="logBPnd"),
set_prior("normal(0, 0.3)", coef="RegionOC",     nlpar="logBPnd"),
set_prior("normal(0, 0.3)", coef="RegionTHA",    nlpar="logBPnd"),
set_prior("normal(0, 0.3)", coef="RegionVSTR",   nlpar="logBPnd"),

set_prior("normal(0, 0.1)", coef="CentreNRU:RegionACC",     nlpar="logBPnd"),
set_prior("normal(0, 0.1)", coef="CentreNRU:RegionAMG",     nlpar="logBPnd"),
set_prior("normal(0, 0.1)", coef="CentreNRU:RegionDBS",     nlpar="logBPnd"),
set_prior("normal(0, 0.1)", coef="CentreNRU:RegionHIP",     nlpar="logBPnd"),
set_prior("normal(0, 0.1)", coef="CentreNRU:RegionINS",     nlpar="logBPnd"),
set_prior("normal(0, 0.1)", coef="CentreNRU:RegionOC",      nlpar="logBPnd"),
set_prior("normal(0, 0.1)", coef="CentreNRU:RegionTHA",     nlpar="logBPnd"),
set_prior("normal(0, 0.1)", coef="CentreNRU:RegionVSTR",    nlpar="logBPnd"),

set_prior("normal(0, 0.1)", coef="CentreNMS:RegionACC",     nlpar="logBPnd"),
set_prior("normal(0, 0.1)", coef="CentreNMS:RegionAMG",     nlpar="logBPnd"),
set_prior("normal(0, 0.1)", coef="CentreNMS:RegionDBS",     nlpar="logBPnd"),
set_prior("normal(0, 0.1)", coef="CentreNMS:RegionHIP",     nlpar="logBPnd"),
set_prior("normal(0, 0.1)", coef="CentreNMS:RegionINS",     nlpar="logBPnd"),
set_prior("normal(0, 0.1)", coef="CentreNMS:RegionOC",      nlpar="logBPnd"),
set_prior("normal(0, 0.1)", coef="CentreNMS:RegionTHA",     nlpar="logBPnd"),
set_prior("normal(0, 0.1)", coef="CentreNMS:RegionVSTR",    nlpar="logBPnd"),

set_prior("normal(0, 0.1 )", coef="CentreNMS", nlpar="logR1"),
set_prior("normal(0, 0.1 )", coef="CentreNRU", nlpar="logR1"),

set_prior("normal(0, 0.1 )", coef="CentreNMS", nlpar="logk2prime"),
set_prior("normal(0, 0.1 )", coef="CentreNRU", nlpar="logk2prime"),

set_prior("normal(0, 0.1 )", coef="CentreNMS", nlpar="logBPnd"),
set_prior("normal(0, 0.1 )", coef="CentreNRU", nlpar="logBPnd"),

set_prior("normal(0, 0.1 )", coef="Age_dec_c", nlpar="logBPnd"),
set_prior("normal(0, 0.1 )", coef="Age_dec_c", nlpar="logk2prime"),

set_prior("normal(0, 0.1)", coef="Age_dec_c", nlpar="logBPnd",
          group="Region", class="sd"),
set_prior("normal(0, 0.05)", coef="DiagnosisMDD", nlpar="logBPnd",
          group="Region", class="sd"),
set_prior("normal(0, 0.05)", coef="TreatmentKetamine", nlpar="logBPnd",
          group="Region", class="sd"),
set_prior("normal(0, 0.05)", coef="TreatmentECT", nlpar="logBPnd",
          group="Region", class="sd"),

set_prior("normal(0, 0.2)", coef="DiagnosisMDD",     nlpar="logBPnd"),
set_prior("normal(0, 0.2)", coef="TreatmentPlacebo",     nlpar="logBPnd"),
set_prior("normal(0, 0.2)", coef="TreatmentKetamine",    nlpar="logBPnd"),
set_prior("normal(0, 0.2)", coef="TreatmentECT",     nlpar="logBPnd"),
set_prior("normal(0, 0.2)", coef="delta_Symptoms", nlpar="logBPnd"),

set_prior("normal(-0.5, 1)", dpar = "sigma"),
```

```
set_prior("normal(0, 0.3)", dpar = "sigma", class="sd", group="PET"),
set_prior("normal(0, 0.1)", dpar = "sigma", class="sd", group="Region:Centre"),

set_prior("normal(0, 0.3)", coef="logRegSize_c", dpar = "sigma", class="b"),
set_prior("normal(0, 0.1)", coef="logRegSize_c", dpar = "sigma",
          group="Centre", class="sd"),

set_prior("normal(1, 0.3)", coef="InjRad_logc", dpar = "sigma", class="b"),
set_prior("normal(0, 0.5)", coef="dur_logc", dpar = "sigma", class="b"),

set_prior("student_t(3, 0, 4)", coef="st_tac:CentreKI_1", dpar = "sigma", class="b")
    ,
set_prior("student_t(3, 0, 4)", coef="st_tac:CentreNMS_1", dpar = "sigma", class="b"
    ),
set_prior("student_t(3, 0, 4)", coef="st_tac:CentreNRU_1", dpar = "sigma", class="b"
    ),

set_prior("student_t(3, 0, 2.5)", dpar = "sigma", class="sds"),

set_prior("lkj(1)", class="cor", group = "ID"),
set_prior("lkj(2)", class="cor", group = "PET:Region"))
```

1.4 Supplementary Materials S4: Simulation Parameters

1.4.1 Global Intercepts

Parameter	Mean
logR1	-0.096
logk2prime	-1.986
logBPnd	0.169
logsigma	-0.815

1.4.2 Individual Deviations

These deviations represent the mean individual deviations. When there are two PET measurements within a single individual, there is only a single deviation from the mean defined for that specific individual.

Standard deviation

Parameter	SD
logR1	0.056
logk2prime	0.115
logBPnd	0.178
logsigma	0.218

Correlation matrix

Parameter	logR1	logk2prime	logBPnd
logR1	1.000	-0.401	0.537
logk2prime	-0.401	1.000	-0.322
logBPnd	0.537	-0.322	1.000

1.4.3 Regional Deviations

For regional deviations, we did not sample from distributions, but rather used the posterior mean deviations for each of the parameters. If we were to sample from distributions instead, we would effectively be simulating a unique set of regions.

Region	logR1	logk2prime	logBPnd	logsigma
FC	0.000	0.128	0.000	-0.797
ACC	-0.079	0.104	0.000	-0.086
AMG	-0.375	-0.031	-0.157	0.207
DBS	-0.103	0.043	0.079	0.963
HIP	-0.162	-0.234	-0.975	-0.158
INS	-0.078	-0.013	-0.040	-0.203
OC	0.148	0.016	0.120	-0.384
THA	-0.006	-0.068	-0.827	-0.365
VSTR	-0.073	0.082	0.479	0.823

1.4.4 PET x Region Deviations

These deviations were defined for the interaction of the PET measurement and the region. For this reason, they accommodate both PET-to-PET variability as well as Region-within-Individual variability.

Standard deviation

Parameter	SD
logR1	0.052
logk2prime	0.065
logBPnd	0.134

Correlation matrix

Parameter	logR1	logk2prime	logBPnd
logR1	1.000	-0.149	0.572
logk2prime	-0.149	1.000	-0.081
logBPnd	0.572	-0.081	1.000

1.4.5 Measurement Error Function

Variation in measurement error, $\log\sigma$, over the duration of the time activity curve was defined with a smooth function and covariates.

The centred smooth function is as follows:

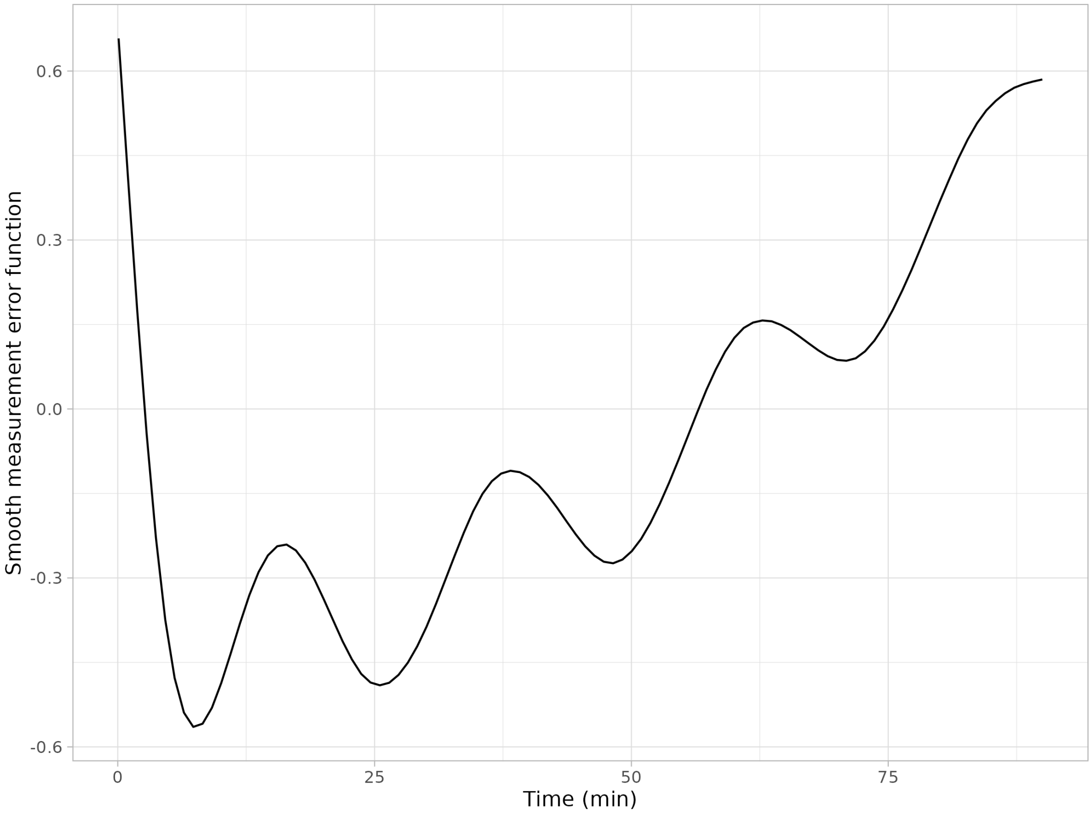

Figure S2. The smooth deviations in the measurement error as a function of TAC time, before accounting for frame duration or region size.

Following addition of measurement error as defined the above function and the region, we also added additional measurement error to account for frame durations by multiplying the centred natural logarithm of the frame duration with the estimated coefficient from the KI dataset of -0.233.

1.5 Supplementary Materials S5: Regional improvements in RMSE of PK Parameters

The following table compares the improvements in the RMSE and correlations with the true values for estimation of the following parameters, comparing NLS with SiMBA with n = 10.

RMSE

Region	NLS	SiMBA	Reduction (%)
R_1			
FC	0.05	0.04	13
OC	0.06	0.05	22
INS	0.06	0.04	34
ACC	0.06	0.04	36
THA	0.06	0.03	40
HIP	0.06	0.03	47
AMG	0.07	0.03	53
VSTR	0.12	0.05	61
DBS	0.14	0.04	69
k_2'			
FC	0.02	0.02	23
OC	0.03	0.02	39
INS	0.03	0.01	46
ACC	0.03	0.02	48
AMG	0.04	0.01	64
THA	0.05	0.02	68
HIP	0.04	0.01	70
VSTR	0.06	0.02	70
DBS	0.08	0.02	79
BP_{ND}			
FC	0.05	0.05	12
INS	0.07	0.06	21
ACC	0.08	0.05	28
AMG	0.18	0.07	60
VSTR	0.34	0.12	64
OC	0.15	0.05	66
DBS	0.48	0.10	79
HIP	0.34	0.03	90
THA	0.34	0.03	91

Correlation

Region	NLS R_1	SiMBA R_1	NLS k_2'	SiMBA k_2'	NLS BP_{ND}	SiMBA BP_{ND}
ACC	0.72	0.82	0.56	0.71	0.97	0.98
AMG	0.59	0.81	0.43	0.70	0.90	0.96
DBS	0.40	0.77	0.25	0.65	0.78	0.94
FC	0.84	0.87	0.71	0.75	0.98	0.99
HIP	0.71	0.88	0.34	0.66	0.79	0.95
INS	0.75	0.85	0.57	0.73	0.97	0.98
OC	0.82	0.86	0.62	0.71	0.97	0.99
THA	0.78	0.88	0.38	0.68	0.85	0.97
VSTR	0.47	0.75	0.37	0.67	0.90	0.96

1.6 Supplementary Materials S6: Parameter Estimation Accuracy for SiMBA estimates only

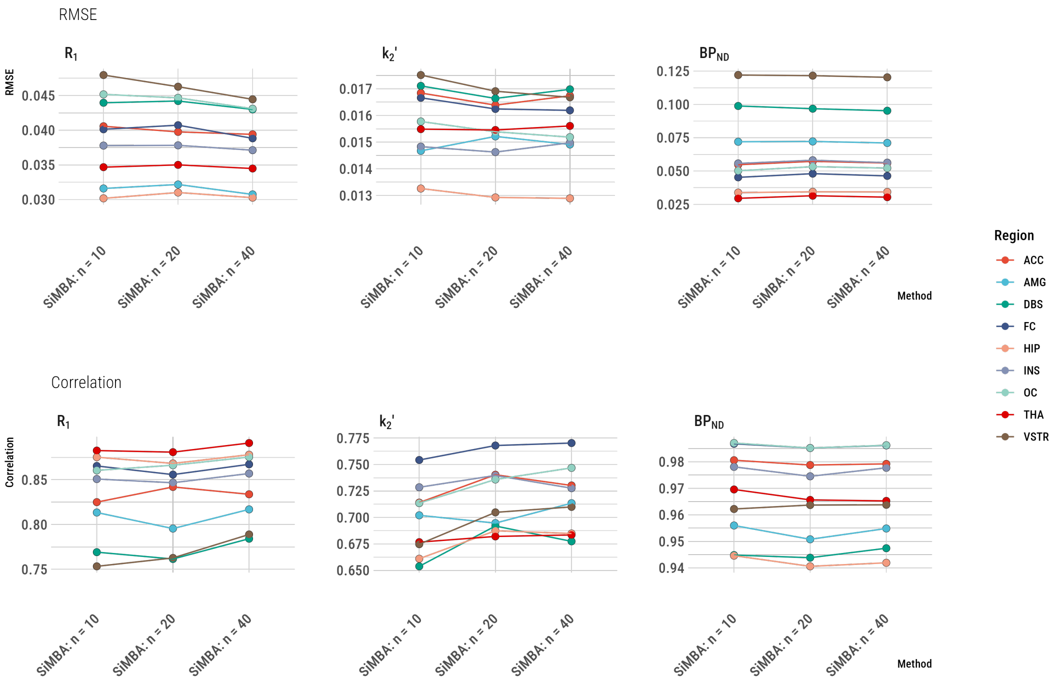

Figure S3. Parameter estimation accuracy assessed by the RMSE and correlation with the true values for SiMBA estimates.

1.7 Supplementary Materials S7: Regional k_2' Estimates

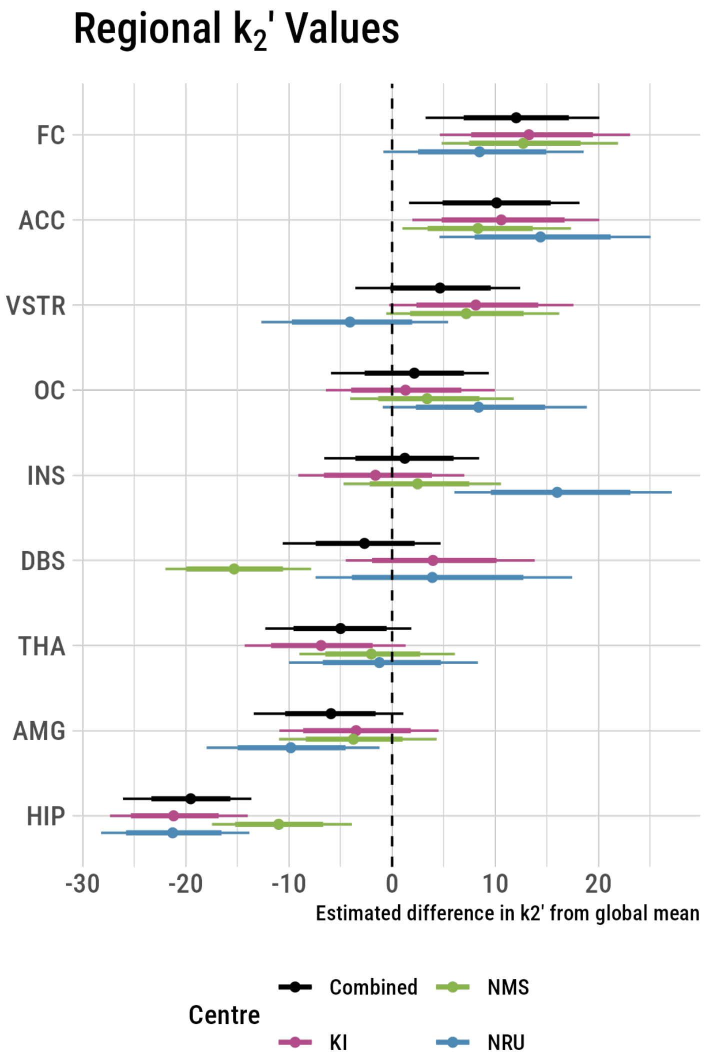

Figure S4. Regional deviations in k_2' are similar between centres.

1.8 Supplementary Materials S8: Correlation Matrices and their Credible Intervals

The individual deviation correlation matrices are shown below.

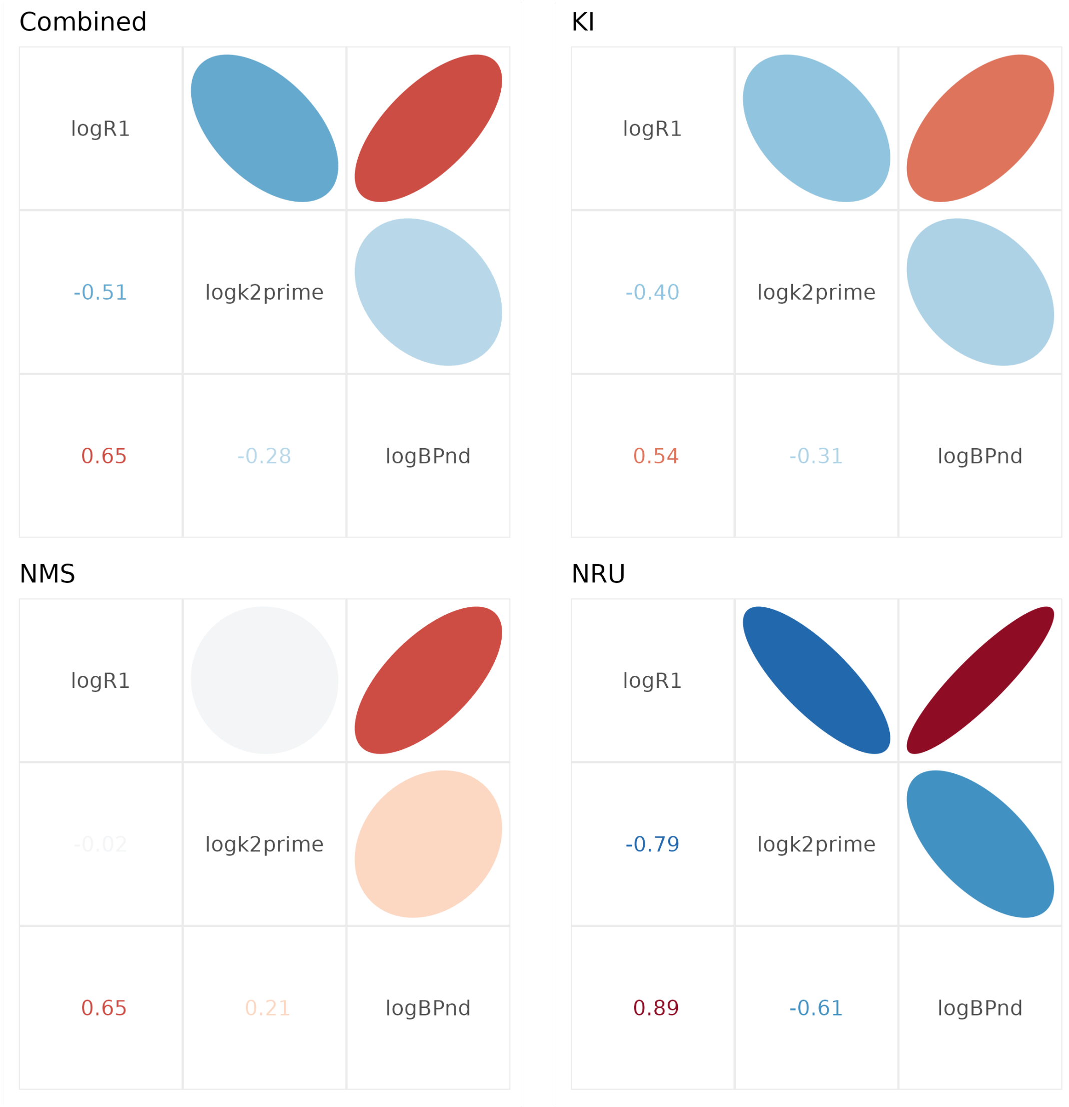

Figure S5. Correlation matrices for individual level deviations for each model.

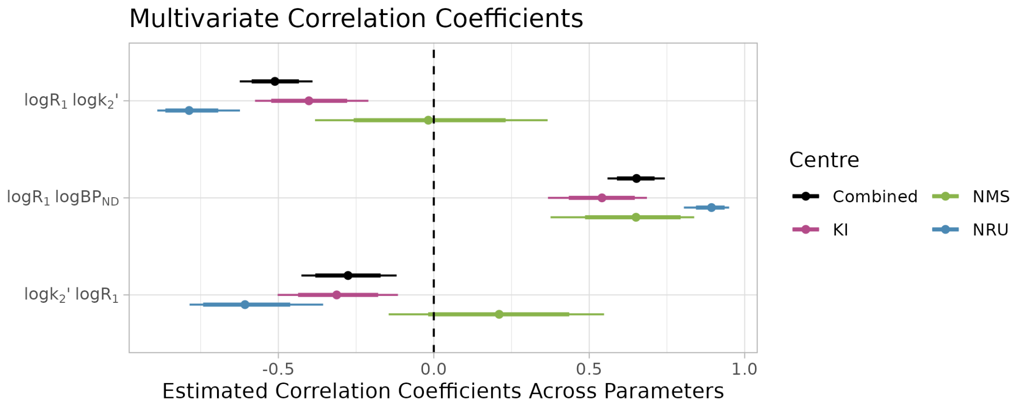

Figure S6. Correlation matrix estimates for individual level deviations with 95% credible intervals.

1.9 Supplementary Materials S9: Age Inferences using LME

The equivalent estimates derived using NLS and LME analysis for Figure 6 are shown below.

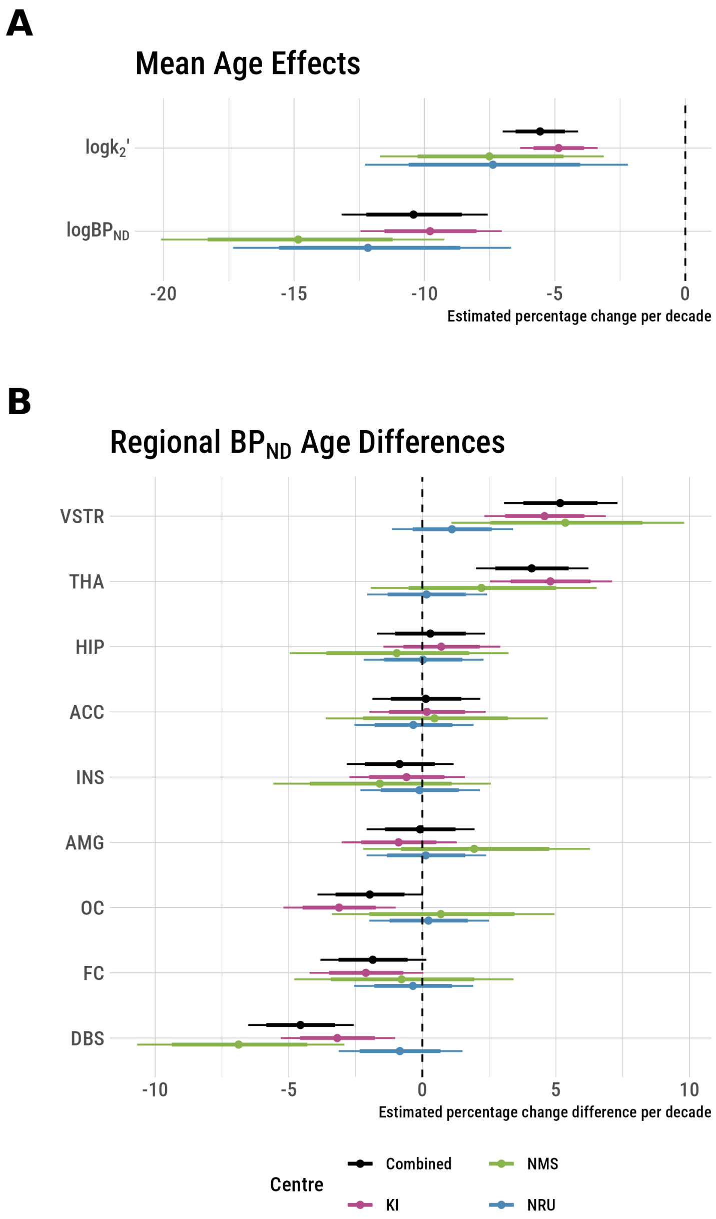

Figure S7. Association of age with PK parameters estimated using NLS and LME. A. Overall mean percentage changes in k_2' and BP_{ND} per decade and their estimates in data from each centre independently as well as the combined dataset. B. Regional deviations from the overall mean age effect from the global mean for each region in the decrease in BP_{ND} per decade. Regional abbreviations are as follows: ACC is anterior cingulate cortex, AMG is amygdala, DBS is dorsal brain stem, FC is frontal cortex, HIP is hippocampus, INS is insula, OC is occipital cortex, THA is thalamus, and VSTR is ventral striatum. Thick error margins represent the 80% credible intervals while the thinner error margins represent the 95% credible intervals.
